# A facile *tert*-butyl nitrite-assisted preparation of deamino graphitic carbon nitride (DA-gCN) as a photocatalyst for the C-H arylation of heteroarenes using anilines as radical source

**DOI:** 10.55730/1300-0527.3605

**Published:** 2023-08-29

**Authors:** Palani NATARAJAN, Begümhan KARAPINAR KOÇ, Önder METİN

**Affiliations:** 1 Department of Chemistry, College of Sciences, Koç University, İstanbul, Turkiye; 2 Department of Chemistry and Centre for Advanced Studies in Chemistry, Panjab University, Chandigarh, India; 3 Koç University Surface Science and Technology Center (KUYTAM), İstanbul, Turkiye

**Keywords:** Deamino graphitic carbon nitride, C-H arylation, photocatalysis, *tert*-butyl nitrite, sustainable chemistry

## Abstract

In pristine graphitic carbon nitride (g-CN), amino groups often function as structural defects that trap photogenerated charges, resulting in low photocatalytic activity as well as reaction with nitrite, aldehyde, etc., ensuing in poor product yield. Without significantly altering the optical characteristics, the removal of amino groups is necessary to increase the photocatalytic activity and structural stability of pristine g-CN. The deamino graphitic carbon nitride (**DA-gCN-5**) was prepared by *tert*-butyl nitrite (TBN)-treatment, characterized and used as a photocatalyst for the radical C-H arylation of heteroarenes using anilines as radical source. Indeed, the photophysical characteristics of **DA-gCN-5** and those of pristine g-CN are very comparable, except that **DA-gCN-5** has a fewer residual amino groups, higher crystallinity, and compressed structure with a different morphology. Moreover, **DA-gCN-5**-catalyzed C-H arylation reaction offers greater product yield in a shorter reaction time compared to that of pristine g-CN in the coupling between heteroarenes and the in situ generated aryl diazonium salts from anilines under visible light irradiation. The amino groups in pristine g-CN absorbed the TBN that was added to convert aniline into the appropriate diazonium ions during the reaction. As a result, deamino graphitic carbon nitride produced by chemical treatment has better photophysical properties and catalytic activity than pristine g-CN. Additionally, this is the first method that uses diazotization reaction for the preparation of deamino graphitic carbon nitride, as far as we are aware.

## 1. Introduction

**Scheme 1:**
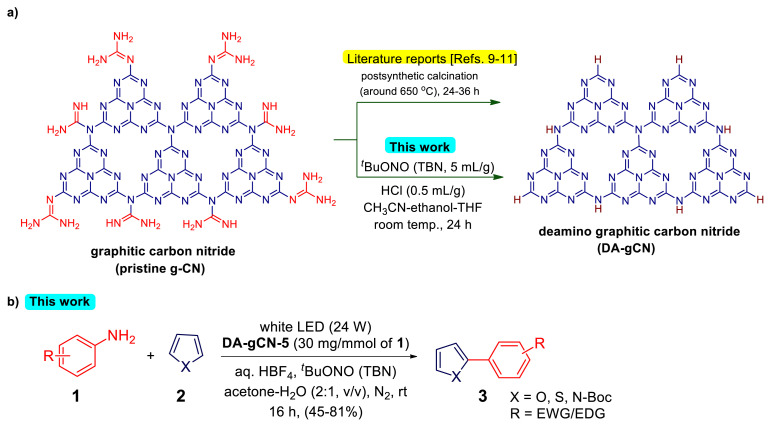
a) An overview of methods used to synthesize deamino graphitic carbon nitride (DA-gCN) from pristine g-CN in the literature [9–11] and this study, and b) the radical C-H arylation of heteroarenes with anilines catalysed by **DA-gCN-5** prepared by our method on TBN-assisted deamination.

Graphitic carbon nitride (g-CN) as a polymeric semiconductor material has recently attracted interest as a potential photocatalyst for various photocatalytic processes [1–3]. It is a visible light responsive substance with a band gap of 2.7 eV [1]. Unlike metal-containing photocatalysts, which require high-cost precursors to be synthesized, g-CN may be made quickly by condensing inexpensive reactants like melamine, dicyandiamide, guanidine, and urea [4–7]. Despite these benefits, the presence of a substantial number of noncondensed amino groups in pristine g-CN severely restricts its usefulness as an efficient photocatalyst [8–9]. These amino groups operate as structural defects that trap photogenerated charges, which results in low photocatalytic activity [8–9]. The postsynthetic calcination process around 650 °C is normally used to remove noncondensed amino groups in pristine g-CN (Scheme 1a). For example, Dai and coworkers synthesized the deamino graphitic carbon nitride via postheating of the g-CN at 630 °C and described its application for the selective photosynthesis of azo- and azoxy-aromatics [10]. Very recently, Zhang and coworkers used the residual heating method to remove the amino-groups in the pristine g-CN and described the photocatalytic performance of calcinated (650 °C) g-CN for rhodamine B degradation, which was up to 7.8 times higher than that of pristine g-CN [9]. Using the catalytic application of deamino graphitic carbon nitride, which is created by postheating the g-CN at 630 °C, our lab has recently demonstrated the successful synthesis of a few organic molecules that will not form in the presence of pristine g-CN as a catalyst [11]. While it yields some promising catalytic performance, the extended calcination at high temperatures (650 °C) causes g-CN to decompose and poses scale-up difficulties [1–3]. Therefore, without significantly altering the optical characteristics, it is crucial to develop a gentle, environmentally benign, and economical process for the deamination of pristine g-CN.

The deamination of aromatic amines is a widely used process in the chemical industry [12]. Despite the fact that there are a variety of deamination processes, deamination of aromatic amines normally involves activating the amino group by diazotization, followed by the replacement of the diazo group with hydrogen [13–15]. It is easy and safe to carry out the conversion through a “one-pot” “one-step” reaction in which the amine is deaminated by a reaction with a source of nitrous acid, such as *tert*-butyl nitrite (TBN), in the presence of a suitable hydrogen donor like tetrahydrofuran, dioxane, or ethanol [16–17]. This method is more appealing than the typical “one-pot” “two-step” textbook procedure, which involves first diazotizing the aromatic amine at low temperature (using sodium nitrite in a mineral acid), and then replacing the diazo group with hydrogen source [13–15]. TBN also functions at room temperature, is gentle, produces no acid waste, and is easy to scale up [16–17]. Nonetheless, the deamination of pristine g-CN and associated compounds using the diazotization technique in general and TBN in particular has not been described yet.

In continuation with our ongoing research on enhancing the photocatalytic performance of pristine g-CN by establishing heterojunctions and doping [7, 18–19], herein we report a TBN-assisted synthesis, characterization, and catalytic efficiency (for the radical C-H arylation of heteroarenes) of the deamino graphitic carbon nitride (**DA-gCN**, Scheme 1b). In fact, when compared to pristine g-CN, the **DA-gCN-5** has a fewer residual amino groups, higher crystallinity, and compressed structure with different morphologies. Moreover, **DA-gCN-5**-catalyzed C-H arylation reaction offers greater product yield in a shorter duration than the pristine g-CN does when coupling in situ produced aryl diazonium salts of aniline with heteroarenes under visible light irradiation. The amino groups in pristine g-CN absorbed the TBN that was added to convert aniline into the appropriate diazonium ions during the reaction. Therefore, even after 48 h of light irradiation, pristine g-CN-catalyzed reactions produced less product than **DA-gCN-5**-catalyzed reactions, as shown in Scheme 1b.

## 2. Experimental section

### 2.1. General

Unless otherwise stated, all reagents and substrates were purchased from commercial sources with the best quality and they were used without further purification. All reactions were carried out under N_2_-gas atmosphere using oven-dried Pyrex glassware unless otherwise specified. All reactions are stirred magnetically unless otherwise specified. All products are known and were characterized by NMR spectra. Chemical shifts are expressed as *δ*-value in parts per million (ppm) and were calibrated using the residual protonated solvent as an internal standard. The peak patterns are indicated as follows: s, singlet; d, doublet; t, triplet; m, multiplet and so on. The coupling constants, J, are reported in Hertz (Hz). Powder X-ray diffraction (PXRD) analysis was performed by using Bruker D2 X-ray diffractometer (Cu Kα, 1.54 Å). Transmission electron microscopy (TEM) was recorded on a Hitachi HT7800 (TEM, 120 kV). Shimadzu 3600 Plus UV-Vis-NIR instrument was used for acquiring the ultraviolet-visible diffuse reflectance spectra (UV-Vis DRS). Photoluminescence (PL) spectra were conducted on Agilent Cary Eclipse PL spectrophotometer with an excitation wavelength of 350 nm. FTIR spectra were conducted on Eclipse FTIR spectrophotometer. The lifetime analysis was conducted with Edinburgh Instrument FLS 1000 spectrometer, 377 nm laser source.

### 2.2. Synthesis of pristine g-CN

Graphitic carbon nitride was prepared by the polycondensation of urea that we previously reported [7]. In essence, 10 g of urea were added to a crucible with a loose lid, heated (calcined) in a muffle furnace at 550 °C for 6 h using a heating rate of 2.5 °C/min, and then allowed to cool naturally at room temperature. The resultant light-yellow graphitic carbon nitride powder sample was denoted as pristine g-CN.

### 2.3. Synthesis of deamino graphitic carbon nitride (DA-gCN)

Four samples of deamino graphitic carbon nitride (**DA-gCN**-X where X = 2, 4, 5, or 6) were prepared using pristine g-CN and 2 mL/1.0 g of g-CN, 4 mL/1.0 g of g-CN, 5 mL/1.0 g of g-CN or 6 mL/1.0 g of g-CN of *tert*-butyl nitrite (TBN) as precursors. The amount of TBN/g of g-CN utilized for its synthesis is indicated by X (number 2, 4, 5, or 6) in the formula **DA-gCN-X** (where X = 2, 4, 5, or 6).

#### Synthesis of DA-gCN-2

Finely ground 500 mg of as-synthesized pristine g-CN powder was taken in a brown glass bottle and dispersed in 30 mL of CH_3_CN-ethanol-THF (1:1:1, v/v, analytical grade) under stirring. Then TBN (1.0 mL) and conc. HCl (250 mL) were added, and stirring was continued for an additional 24 h. Subsequently, the suspension was centrifuged, washed by water followed by ethanol for 3 times each, and dried in a vacuum oven at 80 °C for 12 h. This sample was denoted as **DA-gCN**-2 (2 corresponds to the TBN concentration in mL/1.0 g of pristine g-CN).

#### Synthesis of DA-gCN-4, DA-gCN-5 and DA-gCN-6

To investigate the effect of TBN concentration on the deamination of pristine g-CN, the amount of TBN was changed from 2 mL/1.0 g of g-CN to 4 mL, 5 mL or 6 mL/1.0 g of pristine g-CN. When 2 mL, 2.5 mL, or 3 mL of TBN was used in place of 1 mL of TBN, following the same procedure as the synthesis of **DA-gCN**-2, there were three products that could be obtained. These samples were denoted as **DA-gCN**-4, **DA-gCN**-5, and **DA-gCN**-6.

### 2.4. General procedure for the photocatalytic C-H arylation of heteroarenes

A 10 mL clean and dry borosil glass vial with a magnetic stirring bar were added aniline (**1**, 0.5 mmol), tetrafluoroboric acid (HBF_4_, 0.5 mmol, 90 mL of 48 wt. % in water) and acetone-water mixture (2:1, v/v, 3 mL). Resultant solution was stirred well for 15 min and then TBN (0.6 mmol, 71 mL) was added. Again, reaction mixture was stirred for 15 min at room temperature. Then, heteroarene **2** (10 equiv.) and **DA-gCN-5** (15 mg) were added. Subsequently, under a nitrogen atmosphere, the mixture was irradiated through a plan bottom side of the vial with a 24 W white LED and stirred at room temperature for 16 h. After the reaction, volatiles were removed under vacuum and the residue was diluted with ethyl acetate (5 mL) and filtered to remove the solid catalyst and the solid was washed with ethyl acetate. The entire filtrate was dried on Na_2_SO_4_, filtered, and evaporated to dryness. The crude product was purified by chromatography on silica gel to obtain the pure product **3**. All products are known and were readily characterized by their ^1^H NMR, and ^13^C NMR measurements (vide infra).

#### 2-(4-Chlorophenyl)furan (3a)

Synthesized according to the general procedure described above. Brown solid (67 mg, 75% yield). R_f_ (ethyl acetate:hexane, 5:95, v/v): 0.46. ^1^H NMR (400 MHz, CDCl_3_): δ 7.61 (d, J = 8.4 Hz, 2H), 7.47 (d, J = 1.6 Hz, 1H), 7.34 (d, J = 8.4 Hz, 2H), 6.65 (d, J = 3.2 Hz, 1H), 6.47 (dd, J = 3.2, 1.6 Hz, 1H). ^13^C NMR (100 MHz, CDCl_3_): δ 152.8, 142.3, 132.8, 129.2, 128.8, 124.9, 111.6, 105.4. Spectra data are consistent with those reported in the literature [20].

#### 2-(4-Bromophenyl)furan (3b)

Synthesized according to the general procedure described above. Brown solid (76 mg, 68% yield). R_f_ (ethyl acetate:hexane, 5:95, v/v): 0.44. ^1^H NMR (400 MHz, CDCl_3_): δ 7.57–7.46 (m, 5H), 6.64 (d, J = 3.2 Hz, 1H), 6.48 (dd, J = 3.2, 1.8 Hz, 1H). ^13^C NMR (100 MHz, CDCl_3_): δ 152.8, 142.3, 131.7, 129.6, 125.2, 121.0, 111.7, 105.5. Spectra data are consistent with those reported in the literature [20].

#### 2-(4-Nitrophenyl)furan (3c)

Synthesized according to the general procedure described above. Red solid (77 mg, 81% yield). R_f_ (ethyl acetate:hexane, 5:95, v/v): 0.38. ^1^H NMR (400 MHz, CDCl_3_): δ 8.23 (d, J = 9.0 Hz, 2H), 7.77 (d, J = 9.0 Hz, 2H), 7.57 (d, J = 1.4 Hz, 1H), 6.88 (d, J = 3.2 Hz, 1H), 6.56 (dd, J = 3.2, 1.4 Hz, 1H). ^13^C NMR (100 MHz, CDCl_3_): δ 151.7, 146.3, 144.2, 136.4, 124.3, 123.8, 112.4, 108.9. Spectra data are consistent with those reported in the literature [20].

#### 2-(3-Nitrophenyl)furan (3d)

Synthesized according to the general procedure described above. Pale-yellow semisolid (72 mg, 76% yield). R_f_ (ethyl acetate:hexane, 5:95, v/v): 0.37. ^1^H NMR (400 MHz, CDCl_3_): δ 8.50–8.48 (m, 1H), 8.13–8.08 (m, 1H), 7.98–7.94 (m, 1H), 7.57-7.52 (m, 2H), 6.82 (d, J = 3.4 Hz, 1H), 6.53 (dd, J = 3.4, 1.6 Hz, 1H). ^13^C NMR (100 MHz, CDCl_3_): δ 151.6, 148.5, 143.3, 132.2, 129.6, 129.1, 121.6, 118.5, 112.1, 107.2. Spectra data are consistent with those reported in the literature [21].

#### 2-(4-Ethoxycarbonylphenyl)furan (3e)

Synthesized according to the general procedure described above. Pale-yellow semisolid (79 mg, 73% yield). R_f_ (ethyl acetate:hexane, 5:95, v/v): 0.39. ^1^H NMR (400 MHz, CDCl_3_): δ 8.07 (d, J = 8.6 Hz, 2H), 7.73 (d, J = 8.6 Hz, 2H), 7.51 (d, J = 1.6 Hz, 1H), 6.79 (d, J = 3.4 Hz, 1H), 6.52 (dd, J = 3.4, 1.6 Hz, 1H), 4.37 (q, J = 7.0 Hz, 2H), 1.41 (t, J = 7.0 Hz, 3H). ^13^C NMR (100 MHz, CDCl_3_): δ 166.2, 152.8, 143.0, 134.6, 130.1, 128.8, 123.4, 111.9, 107.1, 60.8, 14.3. Spectra data are consistent with those reported in the literature [22].

#### 2-Phenylfuran (3f)

Synthesized according to the general procedure described above. Orange solid (40 mg, 54% yield). R_f_ (ethyl acetate:hexane, 5:95, v/v): 0.45. ^1^H NMR (400 MHz, CDCl_3_): δ 7.68 (d, J = 7.6 Hz, 2H), 7.49 (d, J = 1.6 Hz, 1H), 7.39 (t, J = 7.6 Hz, 2H), 7.29–7.23 (m, 1H), 6.65 (d, J = 3.2 Hz, 1H), 6.47 (dd, J = 3.2, 1.6 Hz, 1H). ^13^C NMR (100 MHz, CDCl_3_): δ 153.8, 142.1, 130.8, 128.6, 127.2, 123.7, 111.5, 104.8. Spectra data are consistent with those reported in the literature [20].

#### 2-(4-Methylphenyl)furan (3g)

Synthesized according to the general procedure described above. Orange solid (36 mg, 45% yield). R_f_ (ethyl acetate:hexane, 5:95, v/v): 0.44. ^1^H NMR (400 MHz, CDCl_3_): δ 7.56 (d, J = 8.0 Hz, 2H), 7.46 (d, J = 1.6 Hz, 1H), 7.19 (d, J = 8.0 Hz, 2H), 6.58 (d, J = 3.4 Hz, 1H), 6.45 (dd, J = 3.4, 1.6 Hz, 1H), 2.36 (s, 3H). ^13^C NMR (100 MHz, CDCl_3_): δ 154.3, 141.6, 137.2, 129.3, 128.3, 123.7, 111.5, 104.2, 21.2. Spectra data are consistent with those reported in the literature [20].

#### 2-(4-Nitrophenyl)thiophene (3h)

Synthesized according to the general procedure described above. Yellow solid (82 mg, 80% yield). R_f_ (ethyl acetate:hexane, 5:95, v/v): 0.36. ^1^H NMR (400 MHz, CDCl_3_): δ 8.22 (d, J = 8.8 Hz, 2H), 7.75 (d, J = 8.8 Hz, 2H), 7.47 (dd, J = 3.6, 1.0 Hz, 1H), 7.44 (dd, J = 5.0, 1.0 Hz, 1H), 7.16 (dd, J = 5.0, 3.6 Hz, 1H). ^13^C NMR (100 MHz, CDCl_3_): δ 146.7, 141.6, 140.5, 128.7, 127.6, 126.1, 125.7, 124.3. Spectra data are consistent with those reported in the literature [23].

#### 2-(4-Ethoxycarbonylphenyl)thiophene (3i)

Synthesized according to the general procedure described above. Pale-yellow semisolid (88 mg, 75% yield). R_f_ (ethyl acetate:hexane, 5:95, v/v): 0.37. ^1^H NMR (400 MHz, CDCl_3_): δ 8.06 (d, J = 8.4 Hz, 2H), 7.68 (d, J = 8.4 Hz, 2H), 7.41 (dd, J = 3.6, 1.0 Hz, 1H), 7.36 (dd, J = 5.0, 1.0 Hz, 1H), 7.12 (dd, J = 5.0, 3.6 Hz, 1H), 4.40 (q, J = 7.0 Hz, 2H), 1.42 (t, J = 7.0 Hz, 3H). ^13^C NMR (100 MHz, CDCl_3_): δ 166.1, 143.2, 138.4, 130.2, 129.1, 128.2, 126.3, 125.4, 124.5, 60.1, 14.3. Spectra data are consistent with those reported in the literature [22].

#### 2-(4-Ethoxycarbonylphenyl)pyrrole-1-carboxylic acid tert-butyl ester (3j)

Synthesized according to the general procedure described above. Brown oil (109 mg, 69% yield). R_f_ (ethyl acetate:hexane, 5:95, v/v): 0.29. ^1^H NMR (400 MHz, CDCl_3_): δ 8.02 (d, J = 8.4 Hz, 2H), 7.42 (d, J = 8.4 Hz, 2H), 7.38 (dd, J = 3.0, 2.0 Hz, 1H), 6.26-6.22 (m, 2H), 4.38 (q, J = 7.2 Hz, 2H), 1.40 (t, J = 7.2 Hz, 3H), 1.39 (s, 9H). ^13^C NMR (100 MHz, CDCl_3_): δ 166.5, 149.1, 138.6, 134.0, 128.9, 128.8, 128.7, 123.4, 115.3, 110.8, 84.1, 60.9, 27.6, 14.4. Spectra data are consistent with those reported in the literature [22].

#### 2-(4-Cyanophenyl)pyrrole-1-carboxylic acid tert-butyl ester (3k)

Synthesized according to the general procedure described above. Brown solid (63 mg, 47% yield). R_f_ (ethyl acetate:hexane, 5:95, v/v): 0.24. ^1^H NMR (400 MHz, CDCl_3_): δ 7.62 (d, J = 8.4 Hz, 2H), 7.46 (d, J = 8.4 Hz, 2H), 7.39 (dd, J = 3.2, 1.8 Hz, 1H), 6.28-6.23 (m, 2H), 1.40 (s, 9H). ^13^C NMR (100 MHz, CDCl_3_): δ 148.8, 138.7, 133.0, 131.3, 129.6, 123.9, 118.9, 116.1, 111.0, 110.4, 84.3, 27.5. Spectra data are consistent with those reported in the literature [24].

### 2.5. Procedure for control experiments

#### Reaction with BHT

A radical scavenger such as 3,5-di-*tert*-butyl-4-hydroxytoluene (BHT, 2.0 equiv) was added to the general procedure described in Section 2.4. After 16 h, the crude reaction mixture was diluted with ethyl acetate, dried using anhydrous Na_2_SO_4_, filtered, and analyzed by GCMS. Analysis of the GCMS data revealed no product formation.

### 2.6. Procedure for DA-gCN-5 isolation and recycle

After the completion of reaction via the general procedure described in Section 2.4, the product was centrifuged, all the solution was sucked out using a syringe with a needle, and then the catalyst was washed with ethyl acetate, acetone, and water. The reusability of the isolated catalyst was investigated by the same procedure described in Section 2.4. The process was repeated up to seven times.

## 3. Results and discussion

**Figure 1:**
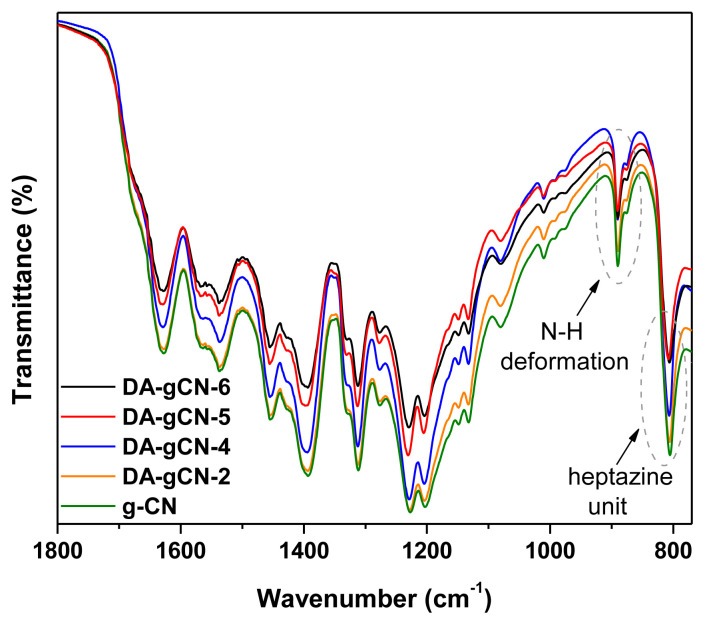
FTIR spectra of **DA-gCN-X** (where X = 2, 4, 5, or 6) and pristine g-CN (for the sake of clarity, 1900–4000 cm^−^^1^ is not shown here).

The Fourier transform infrared (FTIR) spectra of all samples were collected to investigate the change of functional groups. Due to the existence of several functional groups like primary amines, secondary amines, tertiary amines, imines, cyano, and C-H, and their similar frequencies, both the pristine g-CN and **DA-gCN-X** (where X = 2, 4, 5, or 6) have nearly comparable patterns of FTIR spectra (Figure 1). The proportionate characteristic bands at 808 cm^−1^ and 1200–1700 cm^−1^ region, which are attributable to the vibrations of haptazine units and aromatic C-N heterocycles, respectively, TBN merely reacted with the terminal functional groups without modifying the basic aromatic building blocks [4]. Additionally, the broad band at 2900–3420 cm^−1^ could be assigned to the stretching vibrations of the aromatic C-H, O-H, and N-H groups [5]. While the FTIR spectra of **DA-gCN-X** (where X = 2, 4, 5, or 6) and pristine g-CN are identical, some noticeable differences in the N-H deformation area (850–900 cm^−1^) could be seen, which strongly suggested the removal of terminal amino groups [7].

**Figure 2:**
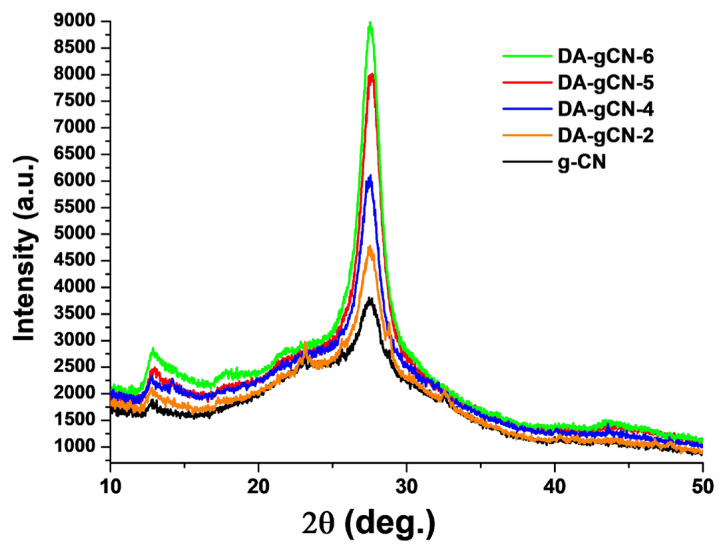
The XRD-pattern of **DA-gCN-X** (where X = 2, 4, 5, or 6) and pristine g-CN.

The XRD patterns were collected to evaluate the crystallinity of **DA-gCN-X** (where X = 2, 4, 5, or 6). Figure 2 shows the XRD patterns of **DA-gCN-X** (where X = 2, 4, 5, or 6) and pristine g-CN. Two peaks at 2θ = 12.8° and 27.5°, respectively, in the patterns of **DA-gCN-X** (where X = 2, 4, 5, or 6) and pristine g-CN reflect the (100) and (002) crystal planes, cf. JCPDS 87-1526 for g-CN [25]. It is apparent that the TBN-treatment did not result in any lattice deformation, which is consistent with the FTIR results (Figure 1). Nevertheless, the intensity of the characteristic peak assigned to (002) facet gradually increases with the increase of TBN concentration, which may be due to regularity caused by deamination. Furthermore, compared with pristine g-CN, the full width at half-maximum of **DA-gCN-X** (where X = 2, 4, 5, or 6) samples peak assigned to (002) facet markedly narrowed, indicating their higher crystallinity. This effect, which was also seen in a kind of extremely crystalline g-CN described in the literature, may have been brought on by the compression of the interlayer distance forced on by van der Waals contact between the (002) layers [26]. Therefore, the overall diffraction intensity and crystallinity of **DA-gCN-X** (where X = 2, 4, 5, or 6) are higher than that of pristine g-CN. In other words, **DA-gCN-X** (where X = 2, 4, 5, or 6) contain less terminal amino groups than pristine g-CN, which has uncondensed packing because of the existence of several directional N-H···N hydrogen bonds through amino groups [26].

**Figure 3:**
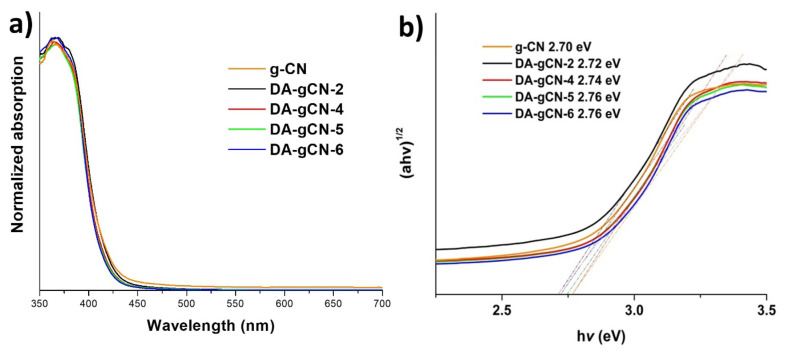
**a)** UV-Vis DRS spectra and **b)** Tauc plot of **DA-gCN-X** (where X = 2, 4, 5, or 6) and pristine g-CN.

The light absorption and band-gap energy of **DA-gCN-X** (where X = 2, 4, 5, or 6) and pristine g-CN were estimated by UV-Vis DRS measurements. In contrast to pristine g-CN, whose absorption edge is located at 451 nm, the absorption edges of **DA-gCN-2**, **DA-gCN-4**, **DA-gCN-5**, and **DA-gCN-6** were at 446 nm, 443 nm, 440 nm, and 439 nm, respectively, which were all located in the visible region (Figure 3a). With the increase of TBN concentration, the absorption edges of the samples had a tendency of blue-shift, which could be ascribed to the loss of terminal amino groups (auxochromes) [15]. It is interesting to note that, consistent with PXRD and FT-IR results, the haptazine core has not been damaged, as shown by the identical pattern of spectra of **DA-gCN-X** (where X = 2, 4, 5, or 6) and pristine g-CN (Figure 3a). To determine the band gap (Eg), Kubelka-Munk model was used to obtain the Tauc plots of **DA-gCN-X** (where X = 2, 4, 5, or 6) and pristine g-CN [7, 18]. As shown in Figure 3b, by extrapolating the straight line to the x-axis, Eg of **DA-gCN-2**, **DA-gCN-4**, **DA-gCN-5**, **DA-gCN-6** and pristine g-CN were determined as 2.72 eV, 2.74 eV, 2.76 eV, 2.76 eV, and 2.70 eV, respectively. Basically, the band gaps of **DA-gCN-X** samples tended to increase with the increase of TBN concentration, which was consistent with the results of DRS (Figure 3a) and the loss of amine auxochromes [15].

**Figure 4:**
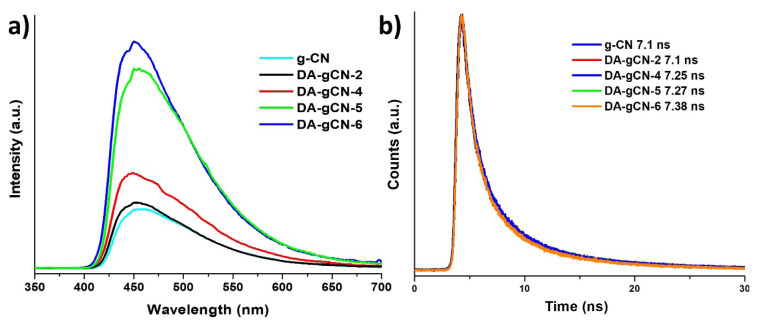
**a)** PL and **b)** time-resolved PL (TRPL) spectra of **DA-gCN-X** (where X = 2, 4, 5, or 6) and pristine g-CN measured using excitation wavelength of 350 nm.

To investigate the removal of amino groups in **DA-gCN-X** (where X = 2, 4, 5, or 6) compared to pristine g-CN, photoluminescence (PL) measurements were also performed. It has been reported that the presence of amino (−NH_2_) groups known to quenching the intensity of the PL peak via photoelectron transfer (PET) processes [27]. The PL spectra (Figure 4a) of pristine g-CN and **DA-gCN-X** (where X = 2, 4, 5, or 6) were measured under identical conditions with the same amount of material, using the excitation wavelength of 350 nm at room temperature. In general, all samples are PL-active (Figure 4a), and fluorescence emission peaks appeared between 420 and 600 nm, indicating that the heptazine core has not been destroyed by TBN treatment and the excited electrons transfer from π* to n orbitals. However, in terms of the PL-intensity, the intensity of the PL peaks was lowest for pristine g-CN and somewhat higher for **DA-gCN-2** and **DA-gCN-4**. Peak intensities for **DA-gCN-5** and **DA-gCN-6** increased significantly while compared to that of pristine g-CN and **DA-gCN-X** (X = 2 and 4). The enhanced PL intensity of **DA-gCN-5** and **DA-gCN-6** clearly indicate that there is loss of amino (NH_2_) groups in these samples [27]. This is in good agreement with the results of FTIR, PXRD, and UV-Vis described above. It was further confirmed by the time-resolved PL (TRPL) lifetime measurements (Figure 4b). As expected, **DA-gCN-5** and **DA-gCN-6** have highest average lifetime with 7.27 ns and 7.38 ns, respectively, compared to pristine g-CN that has average lifetime of 7.10 ns (Figure 4b) [1–2].

**Figure 5:**
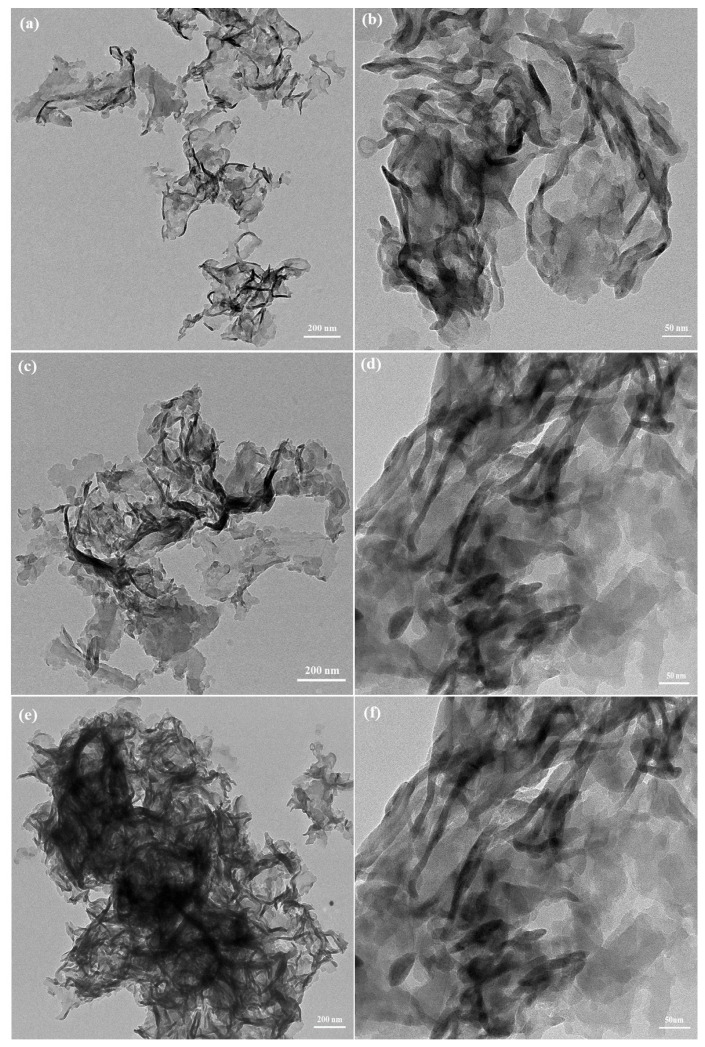
TEM images of pristine g-CN (a and b), **DA-g-CN-4** (c and d) and **DA-g-CN-5** (e and f).

The morphologies of the **DA-gCN-4**, **DA-gCN-5** and pristine g-CN were investigated by transmission electron microscope (TEM) analysis. As shown in Figure 5, the pristine g-CN is composed of thinner sheets while **DA-gCN-4** and **DA-gCN-5** have an irregular thick sheet. Basically, the layer became thicker after deamination or TBN treatment [26]. The compression of the interlayer distance caused by the van der Waals contact between the layers of **DA-gCN-4** and **DA-gCN-5**, as compared to pristine g-CN, may be the cause of this phenomenon. It is in good agreement with the PXRD data.

We then investigated the photocatalytic activity of the deamino graphitic carbon nitride **DA-gCN-X** (where X = 2, 4, 5, or 6) toward the C-H arylation reaction using 4-chloroaniline (**1a**) and furan (**2a**) as model substrates (Table 1). Remarkably, after examining a number of reaction parameters, we discovered that the reaction could produce the desired product **3a** with 75% yield under the following reaction condition: **1a** (64 mg, 0.5 mmol, 1.0 equiv.), **2a** (362 μL, 5.0 mmol, 10.0 equiv.), *tert*-butyl nitrite (TBN, 71 μL, 0.6 mmol, 1.2 equiv.), tetrafluoroboric acid (HBF_4_, 90 μL of 48 wt. % in water, 0.5 mmol, 1.0 equiv.), **DA-gCN-5** (15 mg), acetone-water mixture (2:1, v/v, 3 mL) and 24 W white LED under a nitrogen atmosphere at room temperature for 16 h (Table 1, entry 1). This reaction condition has been named as standard condition (Table 1, entry 1). Control experiments revealed that reactions in acetone and DMSO produced **3a** in moderate yields, while reactions in ethanol, acetonitrile, THF, and water produced low yields (Table 1, entries 2–7). It is significant to note that acetonitrile (CH_3_CN) is a well-known hydrogen source for quenching aryl radicals [15]. The reaction was significantly accelerated by the addition of water to acetone and DMSO, and the best acetone-water ratio 2:1 (v/v) was established (Table 1, entries 8–10). This finding might be explained by the fact that the presence of water could enhance the solubility of the in situ generated aryl diazonium salt, and it keeps the catalyst dispersed over the reaction mixture [19]. The evaluation of other catalyst such as **DA-gCN-6** gave the expected product **3a** in 76% yield (Table 1, entry 11). In contrast, using **DA-gCN-2**, **DA-gCN-4** and pristine g-CN as the photocatalysts gave **3a** in 49%, 60%, and 31% yield, respectively (Table 1, entries 12–14). The complete removal of amino group and high crystallinity of **DA-gCN-5** and **DA-gCN-6** might account for the highest catalytic activity among the four **DA-gCN-X** (where X = 2, 4, 5, or 6) catalysts as well as pristine g-CN. In other words, the amino groups in pristine g-CN, **DA-gCN-2** and **DA-gCN-4** absorbed the TBN that was added to convert aniline into the appropriate diazonium ions during the reaction [13]. Therefore, in all subsequent reactions, **DA-gCN-5** has used as the catalyst unless otherwise specified. The light sources were also examined, and a somewhat low yield of **3a** was obtained when reaction mixture was exposed to blue LED or green LED radiation (Table 1, entries 15–16). Nevertheless, in the dark, there was no reaction. Afterwards, we tested with other acid additives including trifluoroacetic acid and *para*-toluenesulfonic acid. Both produced lesser product **3a** than tetrafluoroboric acid did (Table 1, entries 17–18 and 1). However, when acid was absent, the yield was significantly reduced (26%; see Table 1, item 19). Likewise, under an oxygen environment or an open flask atmosphere, low product (**3a**) yield was observed (Table 1, entries 20–21). Eventually, the survey on the loadings of catalyst **DA-gCN-5** and **2a** clearly showed that 30 mg of catalyst (for 1.0 mmol of aniline **1**) and 10 equiv. of heteroarene (**2**) were adequate (Table 1, entries 22–26). Yet, in the absence of a photocatalyst, no reaction could be seen (Table 1, entry 27).

Thereafter, employing a various heteroarenes (**1**) and anilines (**2**), the substrate scope of the **DA-gCN-5**-photocatalyzed C-H arylation process was investigated under the optimal reaction conditions (Table 1, entry 1). The results are provided in Table 2. The furan (**2a**) with aniline and its derivatives having electron-acceptor substitution (−Cl, −Br, −NO_2_, or −COOC_2_H_5_, **3a-3e**) were generally found to be more effective for product formation than ones with electron-donor substitution (CH_3_, **3g**). This could be explained by the fact that their aryl diazonium salts have lower reduction potentials than those containing electron-withdrawing groups [19]. Subsequently, using certain anilines, we examined the scope of other heteroarenes such as thiophene and N-Boc protected pyrrole. In essence, every reaction proceeded smoothly, producing the appropriate products **3h-3k** in good yield (Table 2). It is important to note that this photocatalysis tolerated a variety of functional groups, including nitro, ester, and halogen groups, which is helpful for further synthetic modification [11].

**Figure 6:**
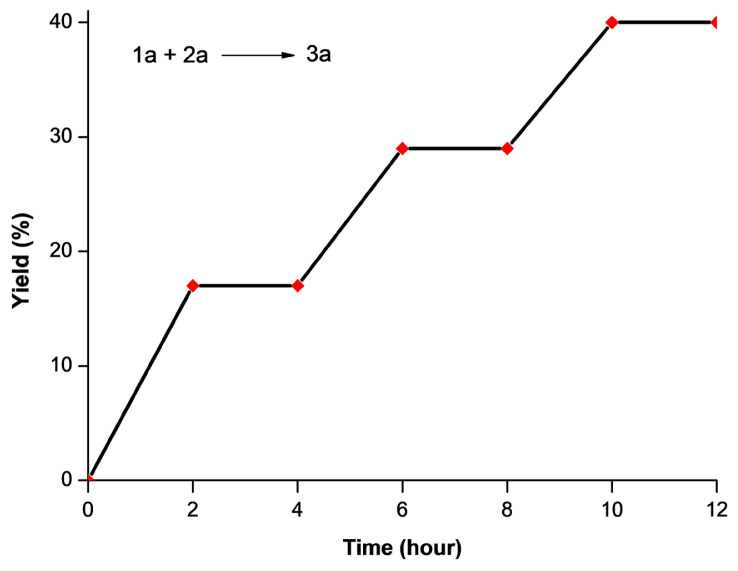
Results of the visible light on-off experiment.

We conducted a light on-off experiment to evaluate if the reaction includes a radical chain mechanism as well as the time profile of the photocatalytic reaction towards the synthesis of **3a** from **1a** and **2a** (Figure 6). The result reveals that in the absence of light, the reaction was completely stopped (no product formation), and that continuous exposure to visible light is necessary for this reaction.

**Figure 7:**
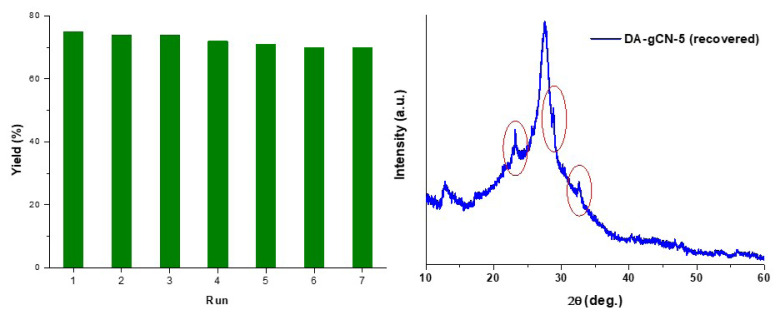
Results of the recyclability of **DA-gCN-5** under the optimal reaction condition for the synthesis of **3a** from **1a** and **2a** (left) and the XRD of recovered **DA-gCN-5** (right).

The development of sustainable processes largely depends on the stability and reusability of heterogeneous photocatalysts [7]. It was important to note that the **DA-gCN-5** catalyst is highly stable and comparable to pristine g-CN. The catalyst may be recovered by centrifugation under the standard reaction condition for the synthesis of **3a**. According to the results, **DA-gCN-5** maintains its catalytic activity for at least seven cycles with just a slight decrease (<2%) after each cycle (Figure 7). The XRD analysis of the regenerated catalyst after seventh cycle was also conducted (Figure 7). Although there are some extra peaks (circles in red in Figure 7) that could be caused by partial structural destruction, no discernible overall structural modifications were observed.

A set of control experiments were then carried out to evaluate the possible reaction mechanism. There was no product (**3a**) when the reaction was carried out in the dark, proving that the reaction requires light (Figure 6). Additionally, no product **3a** was seen in the model reaction when **DA-gCN-5** was absent (Table 1, entry 27), indicating the necessity of the photocatalyst. Next, when radical inhibitor 3,5-di-*tert*-butyl-4-hydroxytoluene (BHT, 2.0 equiv.) was added to the reaction, it was totally inhibited, suggesting that the reaction proceeds through a radical pathway. By employing CH_3_CN as both a hydrogen source and a solvent for the reaction, the radical route of the reaction was further confirmed. Since only 10% of the desired **3a** was produced by the reaction of **1a** and **2a** in acetonitrile (CH_3_CN) medium (Table 1 and entry 5). It is significant to note that TEMPO (2,2,6,6-tetramethylpiperidinoxy radical), a well-known radical quencher, was oxidized by *tert*-butyl nitrite (TBN), resulting in 1-oxopiperidin-1-ium, which prohibits TEMPO usage in the employed conditions of a radical quenching reaction [28].

**Scheme 2:**
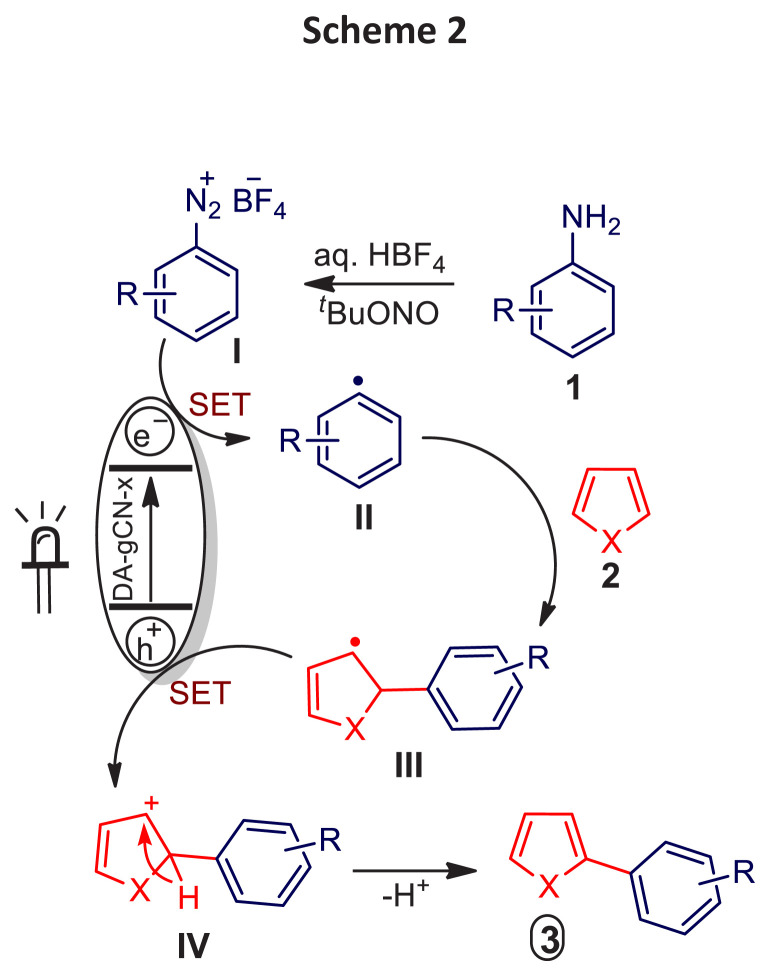
A plausible reaction mechanism.

Based on the control experiments (Table 1) and previous literature reports [29–30], a plausible reaction mechanism is shown in Scheme 2. When exposed to visible light, **DA-gCN-5** is initially excited and goes through a single electron transfer (SET) to produce aryl radical **II** from diazonium salt (**I**) in-situ produced from aniline (**1**). Adding **II** to heteroarene **2** generates the corresponding radical **III**, which is oxidized by the valence band of **DA-gCN-5** into a carbocation intermediate (**IV**, Scheme 2). The required heterobiaryl (**3**) is subsequently produced by deprotonating the unstable carbocation intermediate (**IV**), see Scheme 2.

## 4. Conclusion

As mentioned already, amino groups in pristine graphitic carbon nitride often function as structural defects and leads to low photocatalytic activity and poor product yield. In this regards, postsynthetic calcination process around 650 °C has traditionally been used for the synthesis of deamino graphitic carbon nitride. Due to some limitations of calcination process, at first time, we developed a chemical method using TBN as diazotization agent for the synthesis of deamino graphitic carbon nitride. Moreover, the photocatalytic activity of synthesized deamino graphitic carbon nitride has been explored for the radical C-H arylation of heteroarenes using anilines as radical source. Indeed, compared to pristine g-CN, synthesized deamino graphitic carbon nitride shows higher catalytic activity and afforded expected heterobiaryls in good yield. We believe that the low catalytic activity of pristine g-CN may be due to the poor crystallinity and consumption of some quantity of TBN used as a diazotization agent for in-situ conversion of anilines to corresponding diazonium salts. Thus, we believe that a mild and room temperature method for making deamino graphitic carbon nitride described here, as well as its use as a metal-free catalyst for the C-H arylation of heterocycles, has a promising future, particularly in the fields of medicinal chemistry and the synthesis of functional materials.

## Figures and Tables

**Table 1:** Selected results of screening the optimal conditions for exploring the photocatalytic activity of **DA-gCN-X** for the C-H arylation reaction^a^.

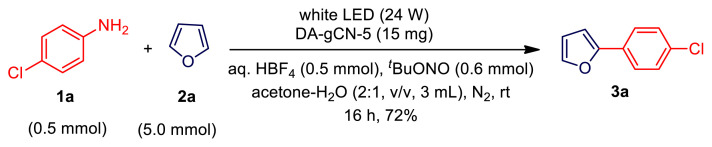		
Entry	Variation of the standard condition a	Yield (%) b
1	standard condition a	75
2	dimethylsulfoxide	47
3	acetone	34
4	ethanol	21
5	acetonitrile	<10
6	tetrahydrofuran	13
7	water	14
8	dimethylsulfoxide-water	51
9	acetone-water (1:1)	58
10	acetone-water (3:1)	66
11	**DA-gCN-6**	76
12	**DA-gCN-2**	49
13	**DA-gCN-4**	60
14	pristine g-CN	31
15	blue LED	67
16	green LED	59
17	*para*-toluenesulfonic acid	61
18	trifluoroacetic acid	17
19	without tetrafluoroboric acid	26
20	under open flask atmosphere	27
21	under oxygen atmosphere	<10
22	**DA-gCN-5** (5 mg)	27
23	**DA-gCN-5** (10 mg)	51
24	**DA-gCN-5** (20 mg)	79
24	**2a** (5 equiv.)	49
26	**2a** (15 equiv.)	76
27	no catalyst (**DA-gCN-5**, 0 mg)	NR

a Standard reaction condition: **1a** (64 mg, 0.5 mmol, 1.0 equiv.), **2a** (362 μL, 5.0 mmol, 10.0 equiv.), *tert*-butyl nitrite (TBN, 71 μL, 0.6 mmol, 1.2 equiv.), tetrafluoroboric acid (HBF_4_, 90 μL of 48 wt. % in water, 0.5 mmol, 1.0 equiv.), **DA-gCN-5** (15 mg), acetone-water mixture (2:1, v/v, 3 mL) and 24 W white LED under a nitrogen atmosphere at room temperature for 16 h.

b GC yield. NR; no reaction.

**Table 2:**
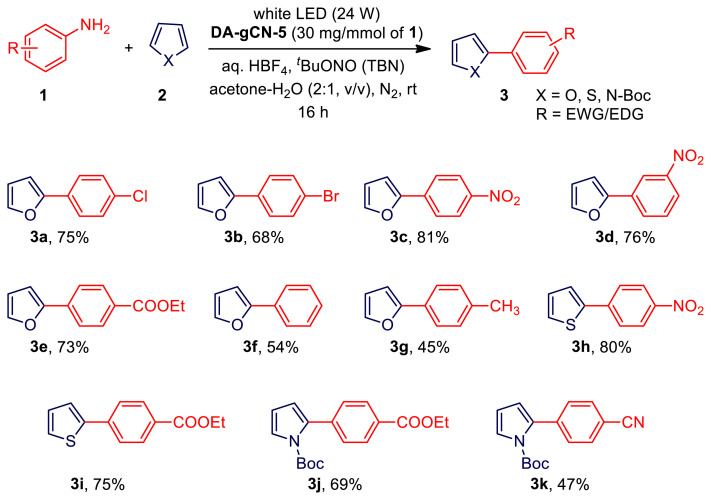
The **DA-gCN-5**-photocatalyzed C-H arylation of heteroarenes with anilines^a^.

a Standard reaction condition: **1** (0.5 mmol, 1.0 equiv.), **2** (5.0 mmol, 10.0 equiv.), *tert*-butyl nitrite (TBN, 71 μL, 0.6 mmol, 1.2 equiv.), tetrafluoroboric acid (HBF_4_, 90 μL of 48 wt. % in water, 0.5 mmol, 1.0 equiv.), **DA-gCN-5** (15 mg), acetone-water mixture (2:1, v/v, 3 mL) and 24 W white LED under a nitrogen atmosphere at room temperature for 16 h.
